# New insights into the mechanochemical coupling mechanism of kinesin–microtubule complexes from their high-resolution structures

**DOI:** 10.1042/BST20221238

**Published:** 2023-08-10

**Authors:** Matthieu P.M.H. Benoit, Byron Hunter, John S. Allingham, Hernando Sosa

**Affiliations:** 1 Department of Biochemistry, Albert Einstein College of Medicine, Bronx, NY 10461, U.S.A; 2 Department of Biomedical and Molecular Sciences, Queen's University, Kingston, ON K7L 3N6, Canada

**Keywords:** cryo-electron microscopy, crystallography, kinesin, microtubule, molecular motors, tubulin

## Abstract

Kinesin motor proteins couple mechanical movements in their motor domain to the binding and hydrolysis of ATP in their nucleotide-binding pocket. Forces produced through this ‘mechanochemical’ coupling are typically used to mobilize kinesin-mediated transport of cargos along microtubules or microtubule cytoskeleton remodeling. This review discusses the recent high-resolution structures (<4 Å) of kinesins bound to microtubules or tubulin complexes that have resolved outstanding questions about the basis of mechanochemical coupling, and how family-specific modifications of the motor domain can enable its use for motility and/or microtubule depolymerization.

## Introduction

**Figure 1.**
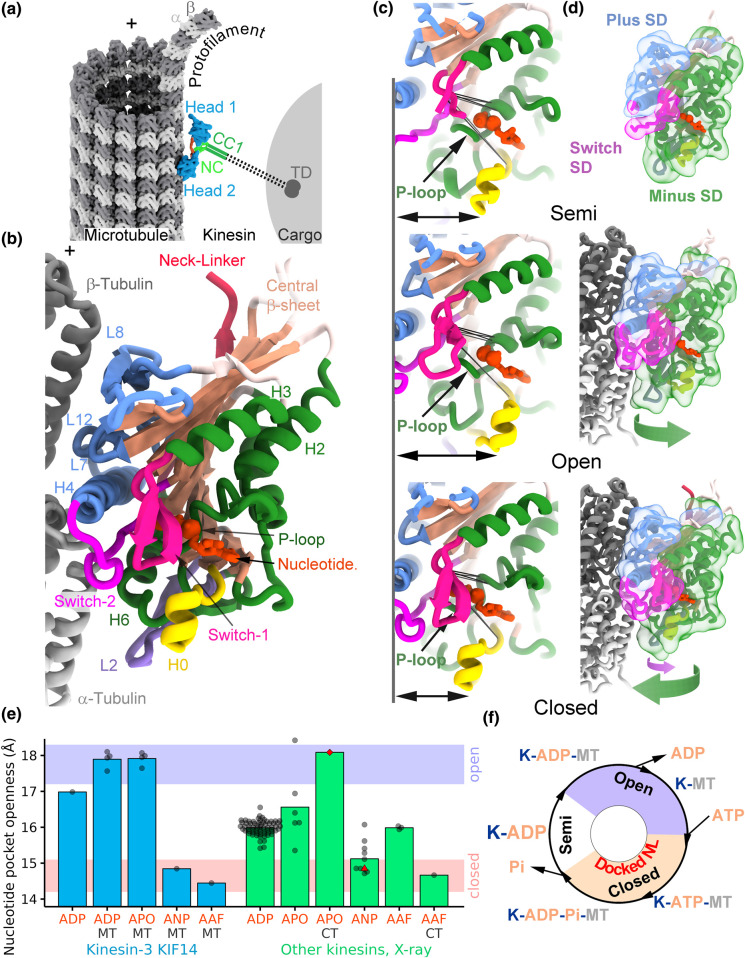
Kinesin motor domain conformations. (**a**) Illustration of a typical kinesin with microtubule plus-end directed motility walking on a microtubule. These kinesins in general contain two polypeptide chains with the motor domain (also called head, blue) located at the N-terminal end. Next in the sequence after the motor domain is the neck-linker (red), followed by an α-helical coiled coil segment, the neck coil (NC, bright green). Following there are additional coiled coil segments (CC1, green) and regions that vary between kinesin families in the number and length of coiled coil segments and other sequence motifs (dashed lines). At the C-terminal end the tail domain (TD) also varies between kinesins. The neck-coil (NC) and other coiled coil dimerization regions place the two motor domains in close proximity and connected through their neck-linkers. (**b**) Cartoon representation of the atomic model of a microtubule bound kinesin motor domain (KIF14, PDBid: 6WWO) with distinct functional and secondary structure regions highlighted in different colors. Loop-8 (L8), loop-12 (L12) and helix-4 (H4) in cornflower-blue; Switch-1 in deep-pink; Switch-2 in magenta; nucleotide in orange-red; helix-2 (H2), helix-3 (H3); helix-6 (H6) and the P-loop in forest-green; helix-0 (H0) in yellow; loop-2 (L2) in medium-purple; central β-sheet in light-salmon; neck-linker in crimson-red; α-tubulin in light-gray; β-tubulin in gray. (**c**) KIF14 motor domain nucleotide-binding pocket close-up views in the semi-closed, open, and closed conformations (PDBid: 4OZQ, 6WWM and 6WWO). Arrows indicate the distance between H4 and H0. Regions colored as in (a). (**d**) KIF14 motor domain subdomains. These are areas within the motor domain that move relative to each other (green and magenta arrows) as the motor transition between the semi-closed, open, and closed conformations. Each subdomain is enclosed by a semitransparent surface. Plus subdomain (Plus SD) in cornflower blue; Switch subdomain (Switch SD) in magenta and minus subdomain (Minus SD) in forest green. (**e**) Nucleotide pocket openness (NPO) is expressed as the average distance between α-carbon atoms of six pairs of highly conserved residues across the nucleotide-binding pocket (black lines shown in paned (**c**)). Residues used are listed in Supplementary Table S1. Each symbol in the plot corresponds to an NPO value calculated from the coordinates of an atomic model and each bar represents the mean of the NPO values in the column. Each column corresponds to a particular kinesin motor domain nucleotide state ADP, Apo, AMPPNP or ADP-AlF_x_ (labeled ADP, APO, ANP and AAF respectively) and the motor domain is microtubule (MT), or curved tubulin (CT) bound. Blue bars correspond to the KIF14 crystal structure and KIF14 microtubule bound cryo-EM structures in the single head bound states. Green bars correspond to the kinesin motor domain crystal structures deposited in the protein data bank except mutant versions and drug bound structures. The NPO values of the first kinesin motor domain high-resolution structures in an open conformation and in the closed catalytic competent conformation are shown respectively with a red diamond and a red triangle symbol (PDBid: 4LNU and 3HQD). The semitransparent blue and red horizontal bars represent the range of values encompassing structures we identified in the open and closed states [16,32,33] (18.3 to 17.2 Å and 15.1 to 14.2 Å, respectively). Values in between these two ranges encompass the semi-closed conformation. The residue pair distances and the NPO values for the structures in this plot and all the ones deposited in the protein databank are listed in Supplementary Table S1. (**f**) Basic mechanochemical cycle for a single motile kinesin motor domain when the neck-linker is free to dock or undock unencumbered by the partner motor domain or by other kinesin-family specific structural elements. Model structure figures were prepared with Blender (panel **a**) and UCSF-ChimeraX [70].

Kinesins are a superfamily of motor proteins that drive a variety of essential cell processes, such as organelle transport, cell division, and cytoskeleton remodeling [1,2]. These motor proteins interact with microtubules tracks, which are filamentous cytoskeletal polymers composed of αβ tubulin heterodimers (Figure 1a). The defining characteristic of the superfamily is the presence of a highly conserved motor domain (Figure 1a,b) that contains binding sites for tubulin and nucleotides and where the energy of ATP hydrolysis is coupled to the generation of mechanical work. Based on the motor domain homology kinesins are grouped into up to 20 families [3,4]. Most kinesins, like the founding member of the superfamily kinesin-1, are motile and move towards the plus end of the microtubule. Kinesisn-14s move in the opposite direction, towards the microtubule minus end and some members of the kinesin-5 family are reported to switch movement directions [5]. Kinesin-13s are microtubule depolymerases and kinesin-8s combine plus end-directed motility and microtubule depolymerization activities [6].

Since the discovery of the founding member of the kinesin superfamily, Kinesin-1 [7,8], much has been discovered regarding the mechanism of action kinesin motor domains using a variety of experimental approaches. The first high-resolution structures of the kinesin motor domain were solved almost three decades ago by X-ray crystallography [9,10] and pseudo-atomic models of the mechanochemical cycle of several kinesins have been built by merging high-resolution (<4 Å) crystal structures of the motor domain with low (∼10–25 Å) and medium resolution (∼4–10 Å) maps of kinesin–microtubule complexes obtained by cryogenic electron microscopy (cryo-EM) [11]. This approach to determining the structure of protein complexes has limitations as the models created are based on the atomic structures of individual proteins, rather than the complex as a whole; the lower the resolution of the complex cryo-EM map, the more the pseudo-atomic models will be biased towards the crystal structures of the individual proteins. As a result, structural changes that occur when the proteins come together to form a complex may not be fully captured. Better than 4 Å resolution is necessary to fully trace the protein polypeptide chain, resolve important regions such as the neck-linker (Figure 1b), determine the positions of side chains and identify the nucleotide species within the nucleotide-binding pocket. This has made it difficult to answer important mechanistic questions, such as the basis of kinesin's ATPase activation by microtubule binding [12], how the two heads of a kinesin dimer coordinate their ATPase cycles, and how a similar motor domain is adapted for motile or microtubule depolymerization activities in different kinesins. However, in the past few years advances in the cryo-EM field, known as the ‘resolution revolution’ [13,14] have facilitated the determination of kinesin–microtubule complex structures at high-resolution (<4 Å).

In this review, we briefly examine how recent high-resolution structural studies of kinesin–microtubule and kinesin–tubulin complexes are improving our understanding of how kinesins link their ATP hydrolysis cycle to force-exerting mechanical movements, and how subtle evolutionary modifications in the motor domain allow different kinesins to perform disparate activities such as motility and microtubule depolymerization.

## Three major conformational states of the kinesin motor domain

High-resolution structures of the motor domain of the motile kinesin-3 KIF14 have been obtained in all the key steps of its ATPase cycle. They include a crystal structure of free KIF14 with ADP in its nucleotide-binding pocket [15], and cryo-EM structures of microtubule-bound monomeric and dimeric KIF14 constructs with ADP, no nucleotide (Apo), the non-hydrolysable ATP analog AMPPNP, and ADP-AlF_x_ as an analog of the ADP-Pi post-hydrolysis transition state [16]. These structures represent the most complete and highest resolution view to date of the mechanochemical cycle of a particular kinesin. They will be used in this review as a reference to describe the conformational changes that occur in a motile kinesin motor domain in response to microtubule binding and the nucleotide species in the nucleotide-binding pocket.

Based on the degree of openness of the nucleotide-binding pocket, the KIF14 motor domain structures can be classified into three major conformations: semi-closed (or semi-open), open and closed (Figure 1c,d). These three conformations are related to distinct steps in the kinesin mechanochemical cycle (Figure 1f). The semi-closed conformation with ADP trapped in the nucleotide pocket represents the microtubule-unbound state. The open conformation represents the ADP release and nucleotide exchange steps occurring after microtubule binding. The closed conformation is the catalytically competent conformation. It is also the conformation where the neck-linker adopts the docked conformation. The extent of nucleotide-binding pocket openness can be expressed as the average distance between highly conserved residues across the nucleotide-binding pocket (black lines across nucleotide pocket in Figure 1c). By this measurement, the three states of the KIF14 motor domain cluster within the ranges indicated in Figure 1e. This measurement can also be used to assess and compare the conformational states of different kinesin motor domain structures. In Figure 1e the nucleotide pocket openness of KIF14 is compared with that of the crystal structures of several kinesins in equivalent nucleotide states (values for all available structures in the Protein Data bank are listed in Supplementary Table S1).

The semi-open conformation of KIF14 was observed in the ADP-bound crystal structure, while the open conformation forms in the microtubule-bound ADP and Apo structures. The closed conformation forms in the microtubule-bound AMPPNP (abbreviated as ANP) and ADP-AlF_x_ (abbreviated as AAF) structures when the complete neck-linker was able to dock into the core motor domain. In the open conformation, the distances between residues involved in nucleotide binding become the largest among the three major conformational states defined. The degree of nucleotide-binding pocket openness of the KIF14 microtubule bound structures is similar to an open conformation first observed in the X-ray crystal structure of Apo kinesin-1 in complex with a DARPin-capped αβ-tubulin dimer [17] (Figure 1e, red diamond symbol). In the closed conformation of microtubule-bound KIF14, the distances between residues involved in nucleotide binding become the shortest among the three major conformational states defined and there is an opening of the hydrophobic pocket formed between β-strand-1 and helix-4 that allows the neck-linker to dock onto the motor domain. Neck-linker docking is the conformational change associated with plus end-directed motility as it results in movement of the microtubule-unbound partner motor domain of a kinesin dimer toward the microtubule plus end [18]. The closed conformation of KIF14 resembles the closed ATP hydrolysis catalytic competent configuration, first observed in the crystal structure of free kinesin-5 Eg5 in complex with AMPPNP [19] (Figure 1e, red triangle symbol). These KIF14 structures, corresponding to both microtubule-bound and unbound states, as well as different nucleotide states, define the complete mechanochemical cycle of a motile kinesin motor domain (Figure 1f).

The transition between the three KIF14 motor domain conformations involve the coordinated movement of multiple regions, which can be approximated as relative movements between three distinct subdomains. We refer to these subdomains as the plus, minus, and switch subdomains (Figure 1d). The plus and minus subdomains form part of the microtubule interface, with the plus subdomain closer to the microtubule's plus end and the minus subdomain closer to the minus end. The switch subdomain is located in between and toward one side of the other two subdomains. It contains the kinesin loops 9 and 11, also known as Switch-1 and Switch-2, respectively. These loops are common structural elements with other NTPases, such as myosins and G-proteins, that change conformation in response to the nucleotide species present in the active site [20] . The central β-sheet serves as the backbone connecting these subdomains and has a different degree of twist depending on the conformation of the motor domain. The more twisted conformation is associated with the open conformation and the less twisted with the closed conformation. The transition from the semi-closed to the open conformation involves a clockwise rotation of the minus subdomain when viewed from the microtubule's minus end. The transition from the open to the closed conformation involves a counterclockwise rotation of the minus subdomain and a comparatively smaller movement of the switch subdomain towards the P-loop. The rotation between subdomains is most clearly appreciated by the larger displacement of regions located at high radius from the rotation axis, such as helix-0 (Figure 1c).

Previous low- and medium-resolution cryo-EM studies of microtubule-bound kinesins have reported distinct closed and open-like conformations of the kinesin motor domain, depending on the nucleotide present [21–29]. However, upon inspecting the nucleotide-binding pocket openness values of the pseudo-atomic models generated from these studies, it was found that some of them had values outside the ranges displayed in Figure 1e for the corresponding nucleotide conditions used (see also Supplementary Table S1). Under Apo or ADP-like conditions, the models exhibited a semi-closed conformation similar to the free kinesin-ADP crystal structures, rather than the open conformation observed for the microtubule-bound KIF14 in the ADP and Apo states. Although structural differences are expected due to the specific kinesin or the experimental conditions used, it is also likely that some of the discrepancies are due to resolution differences between the studies and a bias towards the crystal structures of free kinesin fitted into the lower resolution maps. Fully open motor domain conformations were observed for the first time for kinesin-1 in the Apo state in complex with tubulin by X-ray crystallography [17] and with microtubules by cryo-EM at improved resolution (5–6 Å) than previously available [30]. High-resolution (<4 Å) cryo-EM structures of microtubule-bound kinesin-1 [31], kinesin-8 [32] and kinesin-13 [33] in the Apo state all exhibit a fully open conformation, like the Apo KIF14-microtubule complex structures [16]. The high-resolution of these cryo-EM studies reduces or eliminates biases towards the structures of the individual proteins used in the modeling process, thereby establishing the existence of a distinct open conformation of kinesin in microtubule-bound complexes.

The high-resolution structures of KIF14-microtubule complexes with ADP bound provide a key piece of information regarding the mechanism of kinesin ATPase activation by microtubule binding. These structures and the comparison with the free KIF14 ADP crystal structure show that binding to the microtubule opens the nucleotide-binding pocket and that this conformational change is not a consequence of removal of the nucleotide from the active site, as could have been inferred from the Apo structures alone. On the microtubule, the opening of the nucleotide-binding pocket from the semi-closed to the open conformation allows for the exchange of ADP with an ATP molecule in solution and the subsequent progression of the ATP hydrolysis cycle. A recent report on KIF1A in complex with a microtubule also exhibits an open conformation in the ADP-bound state [34], demonstrating that nucleotide-binding pocket opening by microtubule binding is not unique to KIF14 and is therefore likely to be a general mechanism shared by the kinesin superfamily.

Comparing the KIF14 microtubule complex structures in the presence of ADP-AlF_x_ to the crystal structures of other kinesins in the same nucleotide state indicates that this ADP-Pi analog induces a closed motor domain conformation only when bound to the microtubule or curved tubulin, whereas it leads to a more open conformation when unbound (Figure 1e, columns 5 and 11 vs column 10).

## Allosteric communication between the microtubule-binding site and the nucleotide-binding pocket

**Figure 2.**
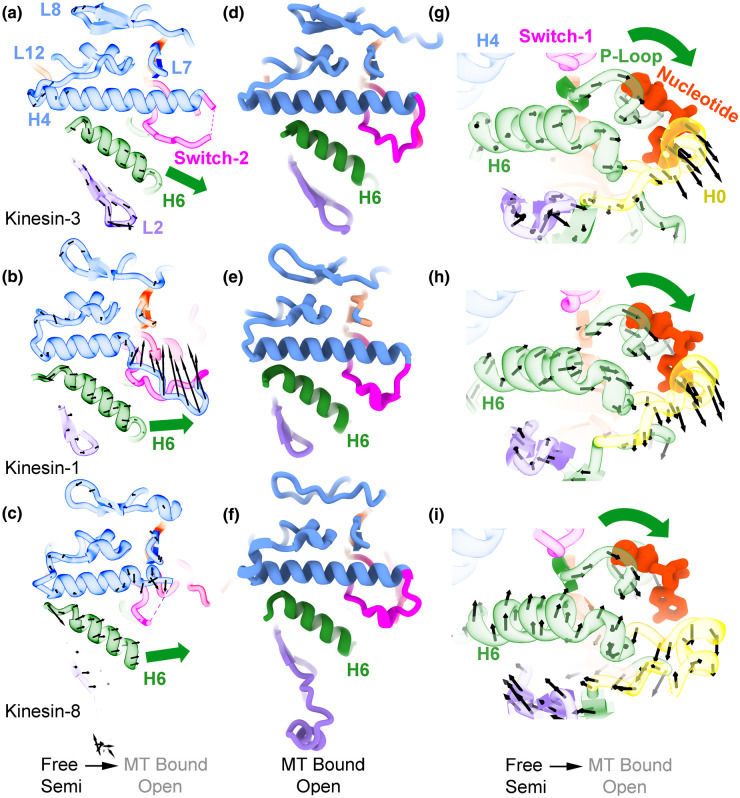
Microtubule-binding-induced conformational changes in the motor domain of three distinct kinesins. (**a**–**c**) View of the microtubule binding region of three ADP-kinesin X-ray crystal structures, kinesin-3 *Mm*KIF14 (**a**), kinesin-1 *Hs*KIF5B (**b**), and kinesin-8 *Ca*KIP3 (**c**). Arrows indicate the direction of structural changes to corresponding microtubule bound open structures (semi-closed to open conformational change). The structures are shown as semitransparent ribbons using the same color scheme as in Figure 1b. Black arrows correspond to the displacement vectors of Cα atoms from the X-ray crystal motor domain structure to the microtubule bound motor domain structure after aligning the plus subdomain (blue colored area) of both structures. For clarity arrows corresponding to the switch-2 region are not shown. (**d–f**) Views of the kinesin microtubule interface in the microtubule-bound open conformation of the same three kinesins shown in **a**–**c**. (**g**–**i**) Same structures and vectors shown in **a**–**c** but viewed from the bottom of the minus subdomain showing part of the microtubule interface and the nucleotide pocket. The PDBid corresponding to the semi-closed and microtubule bound open structures shown or compared in each row are, respectively: 4OZQ and 6WWM (row 1 panels **a**, **d**, **g**), 1BG2 and 8OJQ (row 2 panels **b**, **e**, **h**), 7LFF and 7TQZ (row 3 panels **c**, **f**, **i**).

The tubulin binding interface of the motor domain generally involves three distinct regions, each found in a different subdomain: (i) loops 7, 8 and 12 and helix-4 in the plus subdomain; (ii) switch-2 in the switch subdomain, and (iii) helix-6 in the minus subdomain (Figure 1b,d). Kinesin-8 and 13 form additional tubulin contacts via loop-2 of their minus subdomain, whose effects will be discussed later. The high-resolution structures of kinesin–microtubule complexes reveal in detail the conformational changes occurring with microtubule binding and how these changes are communicated to the nucleotide-binding pocket to modulate the ATPase hydrolysis cycle (Figure 2). The most noticeable differences are observed in or near the switch loops (Figure 2a–f). Parts of these regions are disordered in most crystal structures and become ordered (and visible) in the microtubule bound structures. The extent of these conformational changes varies between kinesins, but it is large enough that it has been previously observed and documented in medium resolution cryo-EM studies [22,26,30]. In some kinesins, there are also movements within the plus subdomain that reposition loop-8, relative to helix-4 (Figure 2b,c). Therefore, binding of all kinesins to the microtubule is an induced fit process but one that differs between kinesins as different kinesins have slightly different interface structures when not bound to the microtubule (Figure 2a–c). Once kinesins bind to microtubules, the overall conformation of the interface becomes more similar (Figure 2d–f).

Several paths of communication between the microtubule and the nucleotide-binding pocket of kinesin have been proposed previously. One involving switch-2 was proposed early on when the first kinesin motor domain structures were solved and the areas of the motor domain at the interface with the microtubule were identified [9,10,35,36]. Based on the crystal structures of kinesin mutants with impaired microtubule activation a chain of conformational changes involving specific residues located in helix-4, switch-2 and switch-1 was proposed [37]. Another path involving loop-7 was proposed based on kinesin crystal structures showing structural differences in this area depending on the position or absence of the Mg^2+^ in the nucleotide-binding pocket [38]. As described above, large conformational changes occur upon microtubule binding in both switch regions and in some cases in helix-4 (Figure 2a–c), supporting proposals where these areas are part of the communication pathway between the microtubule and the nucleotide-binding pocket. On the other hand, changes in the kinesin loop-7 area appears minor in comparison and variable between kinesins, suggesting that this area is unlikely to form the main path of communication between the microtubule and the nucleotide-binding pocket.

An overlooked conformational change induced by microtubule binding that is critical to explain microtubule stimulation of ADP release from kinesin is the relative movement between the three major regions of the tubulin binding interface (Figure 2a–c, green arrows). The movement of helix-6 relative to the other areas of the interface causes a rotation of the minus subdomain relative to the plus subdomain. This movement results in the P-loop region moving away from the switch loops, producing a fully open nucleotide-binding pocket (Figure 2g–i). The open nucleotide-binding pocket facilitates ADP release and nucleotide exchange. Note that the reorganization of the switch loops alone without the subdomain movements would not result in a fully open nucleotide-binding pocket. On the contrary, it would result in a more closed nucleotide pocket as the now organized switch loops without the subdomain movement would wrap around the nucleotide. The structure of the switch loops changes little between the open and closed conformations in the microtubule-bound structures. Mainly, it is the relative movement between subdomains that opens or closes the nucleotide pocket [16]. Thus, we argue that the key conformational change induced by microtubule binding that promotes ADP release is the relative rotation between subdomains.

## Coordination of two kinesins motor domains for processive movement

Coordination of the two motor domains in dimeric motile kinesin such as kinesin -1, -2, -3, and others is thought to be part of the mechanism by which these proteins move processively along microtubules [39,40]. Coordination keeps the mechanochemical cycles of the two motor domains out of phase so that they alternate between microtubule bound and unbound states, and leading and trailing positions, as the molecule walks along the microtubule in a hand-over-hand fashion [41]. Although the evidence supporting this type of coordinated motion is strong, the mechanism of coordination is still unclear and controversial [42,43], in part due to the limited structural evidence that would indicate how one motor domain could affect the activity of the other. This issue is now being addressed through the implementation of specialized cryo-EM image analysis procedures, in particular signal subtraction and symmetry expansion [44,45]. Implementation of these procedures to filamentous structures allow the separation of coexisting structures and structural motifs that do not strictly follow the helical or pseudo-helical symmetry of the microtubule and enable the high-resolution determination of the structure of both motor domains of a kinesin dimer interacting with the microtubule [16].

Studies of dimeric KIF14 constructs in different nucleotide states have shown that in the presence of ADP or in the Apo state only one motor domain is detected bound to the microtubule (single-head-bound) with an open conformation [16]. The partner motor domain is not visible and therefore likely to be very mobile. These structures support previous studies indicating that translocating kinesin-1 dimers form a transient one-head microtubule-bound intermediate with a highly mobile tethered head in the so-called ATP-waiting state [46–48].

**Figure 3.**
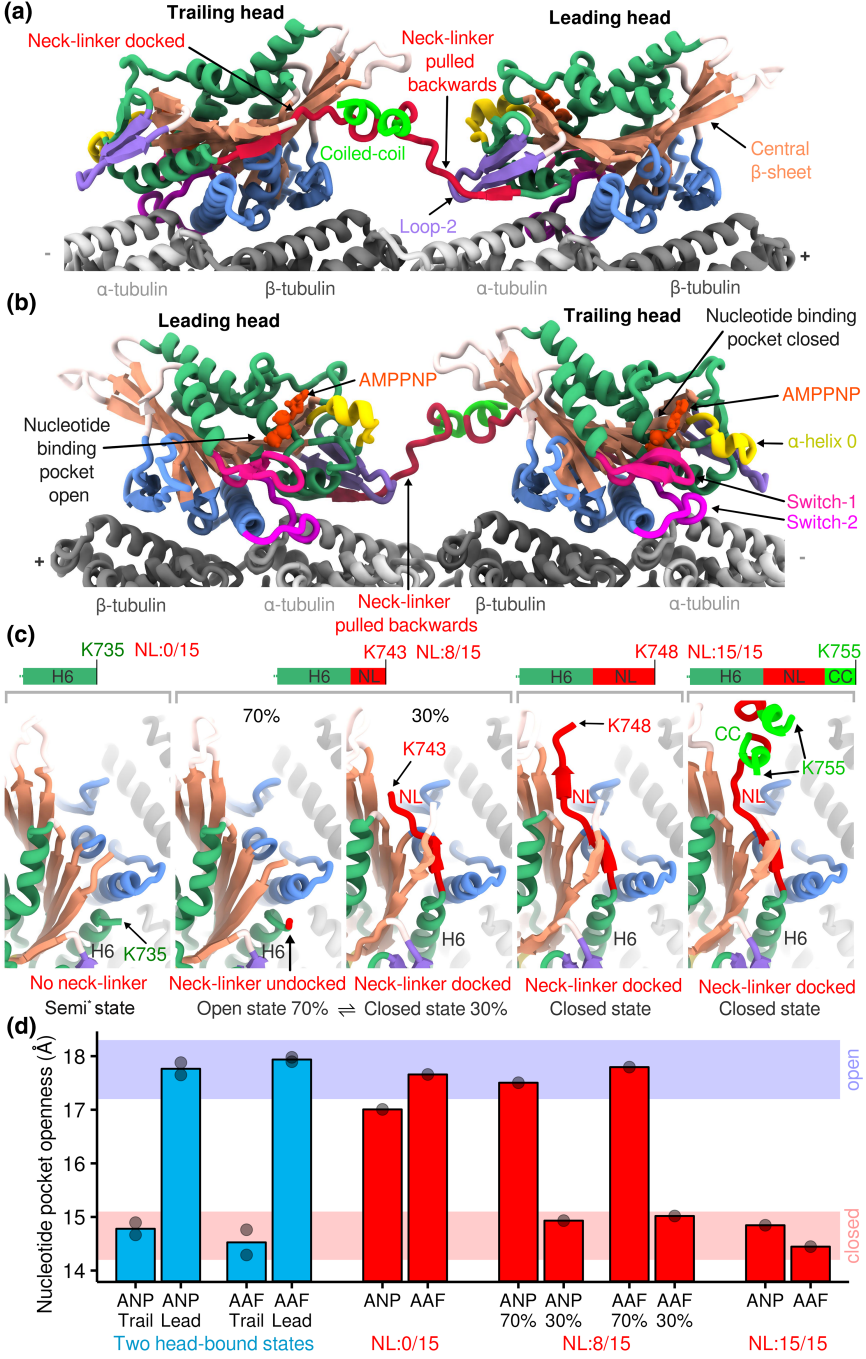
Relationship between neck-linker conformations and nucleotide pocket openness in a motile kinesin-3. (**a** and **b**) Model of the two-head-bound structure of the kinesin-3 *Mm*KIF14 (PDBid: 6WWL), obtained by cryo-EM (resolution of the kinesin part of the map: 3.3 Å). Both heads are AMPPNP bound. Key parts of KIF14 leading and trailing heads and of the microtubule are labeled with the same color scheme as in Figure 1b. Microtubule polarity is indicated with + and − signs. The views in (**a**) and (**b**) are rotated 180° to each other to emphasize respectively the distinct positions of the neck-linkers and the distinct conformations of the nucleotide-binding pockets. (**c**) Comparison of the neck linker position in four microtubule-bound KIF14 constructs (K) bound to AMPPNP and differing by the length of their C-termini. The number of the last residue of each construct as well as the number of neck-linker residues are indicated. K735: no neck-linker, K743 has the first eight of 15 neck-linker residues, K748 has the full neck-linker. K755 has the full neck-linker and the first coiled-coil residues and is a two-heads-bound state on the microtubule. Semi*: The K735-ANP structure (microtubule bound KIF14 with no neck-linker in the presence of AMPPNP, PDBid: 6WWV) was previously called open* but due to its slightly more closed nucleotide-binding pocket than other structures in the open conformation [16], it falls within the semi conformation group according to the NPO boundaries defined in Figure 1. (**d**) Quantification of the nucleotide pocket openness of several KIF14 constructs differing by the length of their C-termini, in both AMPPNP and ADP-AlF_x_. Note that only the constructs with a full neck-linker assume the fully closed state seen in the trailing head of the two heads-bound-states.

In the presence of AMPPNP or ADP-AlF_x_, both motor domains of KIF14 dimers bind to the microtubule at contiguous tubulin heterodimers along a protofilament, forming a two-head-bound state (Figure 3a,b). The two-heads bound structure of KIF14 exhibits a trailing motor domain in the closed conformation with a docked neck-linker and a leading motor domain with an open conformation and undocked, backward oriented neck-linker (Figure 3a). The distinct conformation of the leading and trailing motor domains provides direct structural evidence of coordination between them to keep each motor domain mechanochemically out of sync with its partner.

Despite the presence of nucleotides such as AMPPNP or ADP-AlF_x_ that typically induce the closed conformation, the leading motor domain in the two-heads bound state remains in the open conformation. Similarly, monomeric KIF14 constructs with fully truncated neck-linkers fail to adopt the closed conformation in the presence of AMPPNP or ADP-AlF_x_, while constructs with partially truncated neck-linkers result in a mixed conformational population, with ∼70% adopting the open conformation (Figure 3c,d). These results indicate that a fully docked neck-linker is required to adopt the closed conformation and that the major determinant preventing the leading head of the dimer from adopting the closed conformation is that its neck-linker is pulled out of the docked conformation by the trailing head. The neck-linkers therefore coordinate the activity of the two motor domains keeping their ATPase cycles out of sync. In the two head bound state only the trailing head can have a docked neck-linker and closed conformation while the leading head has an undocked neck-linker and is in the open conformation. For the leading head to achieve the closed conformation and proceed with its ATP hydrolysis cycle the trailing head must detach so that the neck-linker of the leading head can dock. As the neck-linker of the leading heads docks, the trailing head moves forward, and the two heads exchange trailing and leading positions.

**Figure 5.**
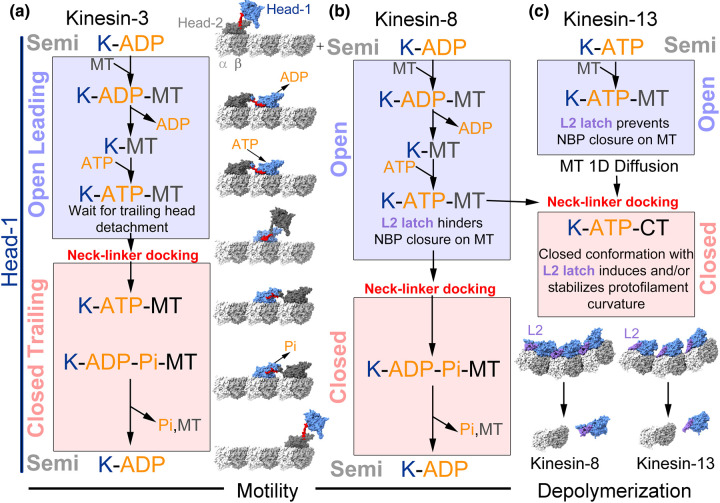
Coordinated kinesin mechanochemical cycles. (**a**) Mechanochemical cycle of one of the motor domains (head-1) of kinesin dimer. The partner motor domain (head-2) has a similar cycle to head-1 but synchronized out of phase so that when head-1 is in the open leading conformation head-2 is in the closed trailing conformation and vice versa. (**b**) Mechanochemical cycle of a kinesin-8 combining motile and microtubule depolymerization activities. When interacting with the microtubule the mechanochemical cycle is similar to the one of motile kinesins (**a**) but the interaction of loop-2 with the microtubule prevents nucleotide-binding pocket closure with ATP binding but it occurs with ATP hydrolysis. At microtubule ends, the loop-2 tubulin interaction in the ATP bound state promotes tubulin curvature and microtubule depolymerization. Whether the two motor domains of kinesin-8 dimer have tightly coordinated mechanochemical cycles as proposed for motile kinesins (**a**) remains to be investigated. (**c**) Mechanochemical cycle of microtubule depolymerase kinesin-13. In this kinesin's unbiased one-dimensional diffusion, rather than unidirectional motility, occurs when interacting with the microtubule lattice. As in the case of kinesin-8s, the interaction of loop-2 with tubulin prevents ATP binding induced closure of the nucleotide-binding pocket when bound to the microtubule lattice. When bound at microtubule ends, ATP binding results in nucleotide-binding pocket closure with stabilization and/or induction of tubulin curvature.

## Modulation of the kinesin mechanochemical cycle for microtubule depolymerization

Kinesin-8 and kinesin-13 motors are unique members of the kinesin superfamily due to their ability to catalyze removal of tubulin subunits from microtubule ends. Kinesin-8 proteins are typically slow, motile microtubule depolymerizers and their primary function is to limit the maximum length of mitotic spindle and cilia microtubules [49]. In contrast, kinesin-13s are non-motile, yet more potent microtubule depolymerizers that mediate large-scale remodeling of interphase microtubules during the entry into mitosis, modulation of microtubule dynamics during mitosis, disassembly of the mitotic spindle at the end of mitosis, and regulate cilia length [50]. While both motors bind the microtubule lattice and can recognize the curved conformation of the tubulin protofilaments at microtubule ends, each motor reaches the microtubule end and interacts with curved tubulin by a different mechanism. Kinesin-8s are plus-end directed motile kinesins while kinesin-13s are reported to bind weakly to the microtubule lattice and undergo unbiassed one-dimensional diffusion [51]. Structural studies suggest that modified minus subdomains (i.e. extended loop-2s and the kinesin-13 neck domain) underly these mechanistic differences and that these modifications are also important determinants of microtubule depolymerase activity itself [32,33,52–55].

**Figure 4.**
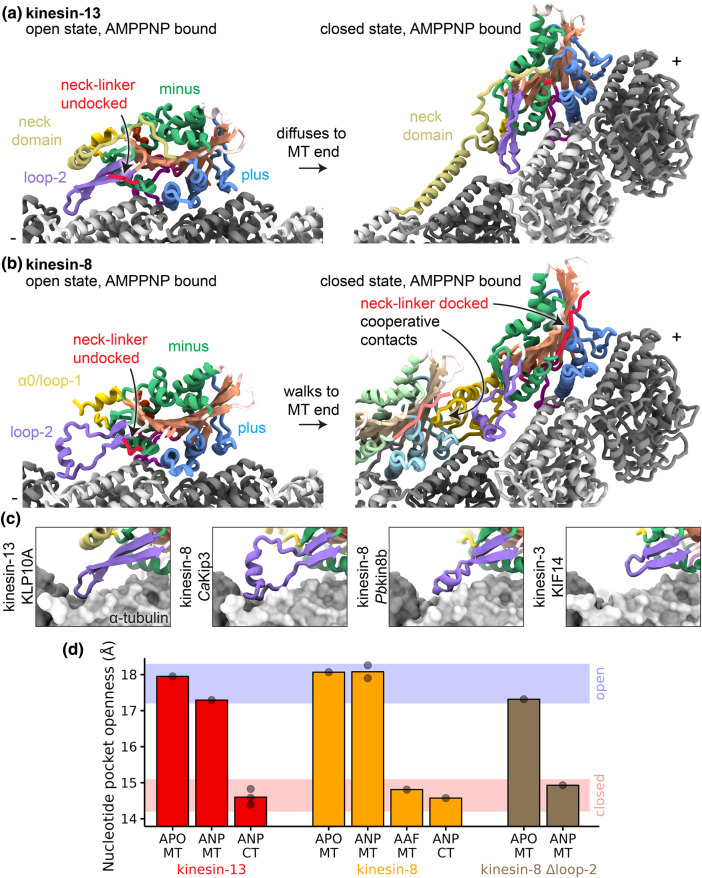
Modulation of the kinesin mechanochemical cycle for microtubule depolymerization. (**a**) Kinesin-13 (KLP10A, PDBid: 6B0L) adopts an open state on the straight microtubule when bound to AMPPNP (left panel). On curved tubulin protofilaments, kinesin-13 (KLP10A, PDBid: 6B0C and KIF2A, PDBid: 6BBN, the latter illustrated) adopts a closed state when bound to AMPPNP (right panel). (**b**) Kinesin-8 (*Ca*Kip3, PDBid: 7TQX) adopts an open state on the straight microtubule when bound to AMPPNP (left panel). On curved tubulin protofilaments, kinesin-8 (*Ca*Kip3, PDBid: 7TR3) adopts a closed state when bound to AMPPNP (right panel). Minus-end kinesin-8 neighbor shown in faded colors to highlight cooperative contacts. (**c**) Close-up views of loop-2 from separate kinesin families to illustrate different loop-2 structures and tubulin contacts. From left to right: KLP10A — PDBid: 6B0L, *Ca*Kip3 — PDBid: 7TQX, *Pb* kinesin 8b — PDBid: 7Z2A, KIF14 — PDBid: 6WWI. (**d**) Nucleotide-binding pocket openness measurements for high-resolution structures of kinesin-13 and kinesin-8 motors. Measurements performed using same residues as Figures 1 and 3.

Early functional and crystallographic studies of kinesin-13s revealed the importance of the family-specific extended loop-2 for microtubule depolymerization [52,53]. High-resolution structures of human KIF2C [54] and KIF2A [55] in complex with tubulin heterodimers and of drosophila KLP10A in complex with microtubules and curved tubulin protofilaments [33] gave the first descriptions of how the kinesin-13 mechanochemical cycle is tuned for depolymerization (Figure 4). These structures revealed how kinesin-13s form additional tubulin contacts through their extended loop-2 and a class-specific neck domain that can occupy an adjacent tubulin subunit (Figure 4a, right panel). Cryo-EM structures of KLP10A on the microtubule [33] showed that in contrast with non-depolymerizing kinesins, the nucleotide-binding pocket of the motor domain remains open in the ATP-bound state on the straight microtubule lattice (Figure 4a,d) but closes when bound to curved tubulin protofilaments [33]. This provided an explanation for the lack of motility of kinesin-13s and how these kinesins are specifically tuned for depolymerization. Presumably this is due to the observed loop-2 microtubule interactions acting as a latch to restrict rotations of the minus subdomain and hindering the nucleotide-binding pocket from closing. It indicates that the loop-2 of kinesin-13 helps coupling the ATPase cycle to the shape of the bound tubulin. As a result, nucleotide pocket closure in the ATP-bound state occurs only after binding to curved tubulin or after bending straight tubulin. In the closed, catalytically active state, the motor interface (aided by loop-2 and the neck domain) can further bend protofilaments to trigger microtubule depolymerization. [33,55].

A structural explanation for how kinesin-8 motor domains combine motility and depolymerization was provided by high-resolution structures of the *Candida albicans* Kip3 motor domain [32] (Figure 4b). Analogous to kinesin-13s, the AMPPNP-containing nucleotide-binding pocket of this motor domain is held open on the straight microtubule lattice by interactions between the kinesin-8-specific extended loop-2 and the microtubule. Accordingly, Kip3 constructs without the elongated loop-2 (Kip3 loop-2 swapped for the shorter kinesin-1 loop-2) adopt the closed conformation with AMPPNP (Figure 4d). Structures of the ADP-Pi state of *Candida albicans* Kip3 showed that interactions between the extended loop-2 of kinesin-8 and the microtubule eventually break, unrestricting rotation of the minus subdomain to allow nucleotide-binding pocket closure and neck-linker docking. This allows the other head to move forward and the motor to walk along the microtubule towards the microtubule end. Upon reaching curved tubulin protofilaments at microtubule ends (Figure 1a), kinesin-8s adopt a pro-depolymerization state when bound to ATP. Here, different from what occurs in the microtubule lattice, the lack of lateral contact of the microtubule protofilaments and its curvature facilitates loop-2 and the minus subdomain to rotate, closing nucleotide-binding pocket and allowing the neck-linker to dock. The presence of curved tubulin protofilaments at the ends of microtubules [56] suggests that depolymerizing kinesins may stabilize these pre-existing curved protofilaments and induce further tubulin curvature [32,33,55,57]. This process leads to microtubule depolymerization as the stabilized curved tubulin protofilaments are unable to form lateral contacts and close the microtubule wall.

In lieu of the kinesin-13 neck domain, kinesin-8-mediated depolymerization appears to be enhanced by cooperative contacts formed between motors residing on adjacent curved tubulins. The cryo-EM tubulin ring-bound structure of Kip3 shows that these contacts involve its extended loop-1 sequence. However, not all kinesin-8s harbor an elongated loop-1, so other mechanisms of cooperativity are likely to be found or could simply arise from the accumulation of bending activities by multiple kinesin-8 on the same protofilament.

The role of kinesin-8's loop-2 in its microtubule depolymerization activity appears partially different from kinesin-13s. In kinesin-13, loop-2 is required for depolymerization activity [52,53,58], whereas Kip3 constructs without an elongated loop have severely impaired but not abolished microtubule depolymerization activity[32,59]. Different kinesin-8s also have different loop-2s (Figure 4c), and they also appear to interact with the microtubule in different manner [32,60] suggesting that this element may modulate motility and microtubule depolymerization activities in a different manner in different kinesin-8s. A summary of the mechanochemical cycles for kinesin-3s, kinesin-8s and kinesin-13s is presented in Figure 5.

## Modulation of the kinesin mechanochemical cycle by elements outside the motor domain

Apart from structural elements inside the motor domain such as the neck-linker and the loop-2 discussed in the previous sections, the activity of kinesins in cells is selectively turned on and off by interactions between the motor domain and elements outside the motor domain such as the neck and tail domains or other accessory proteins such as kinesin binding proteins (KBP) [61]. Recent structural work has provided medium to high-resolution views of the interaction between some of these regulatory factors and the kinesin motor domain.

Kinesin-3s can enter an autoinhibited state where coiled-coil regions following the motor domain, interact with each other preventing dimerization [62]. Crystal structures of the kinesin-3 KIF13 in the uninhibited and inhibited form reveal that one of its coiled-coil regions (CC1) is broken into two short helices that interact with the first coiled-coil segment after the neck-linker (the neck-coil) and the motor domain, preventing dimerization and locking the neck-linker in the docked position [63].

Kinesin-5s are bipolar homotetrameric proteins with two motor domains and two tail domains on each side of the molecule. This structural organization is tailored to their role in cross-linking and sliding mitotic spindle microtubules [5]. Recent cryo-EM studies revealed that the tail domain of the kinesin-5 KLP61F binds to helix-0 of the motor domain specifically in the Apo (i.e. open) conformation. The authors propose that this interaction slows ATP-binding and results in higher kinesin-5 force production [64].

Two recent cryo-EM studies revealed the structures of the kinesin binding protein (KBP) alone and in complex with kinesin motor domains [65,66]. KBP mutations are associated with the neurological disorder Goldberg-Shprintzen syndrome (GOSHS) and this protein is known to bind to 8 of the 45 kinesins present in the human genome [67]. The structures reveal that KBP binds to the microtubule binding interface of the kinesin motor domain to block microtubules binding and induce a large conformational change where the motor domain helix-4 is pulled ∼20 Å away from the motor domain core. The studies also reveal possible areas of KBP that may be involved in binding discrimination among kinesins.

In a tour de force, Han et al. [68] solved the structure of over 70% of the mass of the *Chlamydomonas reinhardtii* cilia central apparatus at high-resolution. This apparatus is a large macromolecular complex comprising the two central microtubules of the cilia axoneme, along with more than 45 distinct proteins organized in 32 nm repeats. Among these proteins there is a kinesin-9 called KLP1, which is essential for normal flagellar motility [69]. The KLP1 molecules were found in the central apparatus structure to form microtubule bound asymmetric dimers with a trailing head in a closed conformation with a docked neck-linker and a leading head with an undocked neck linker in a more open conformation. This configuration bears some resemblance to the previously described KIF14 microtubule bound dimer structures, (Figure 3a,b). However, unlike the KIF14 structures, both heads of the KLP1 structures were reported to contain ADP in the nucleotide-binding pocket. In contrast, for KIF14, the two-head-microtubule bound intermediate only forms with analogs of the ATP or ADP-Pi states [16]. Furthermore, the closed conformation with docked neck-linker, observed in the trailing head of KLP1, is only observed in other kinesins with ATP or ADP-Pi analogs, not with ADP (see Supplementary Table S1). Additionally, the leading head of KLP1 adopts a semi-closed conformation, rather than the fully open conformations observed in the leading head of the two-heads-bound KIF14 structures or in the microtubule-bound ADP structures of KIF14 and other kinesins. It remains to be investigated whether these differences arise from unique properties of KLP1 or if they are induced by the accessory proteins interacting with KLP1 in the central apparatus complex.

## Perspectives

- High-resolution structural determination of kinesins and other cytoskeletal motor proteins bound to their filamentous tracks is essential to understand their molecular action mechanism. Recent advances in cryo-electron microscopy now allows the structure of these complexes to be determined at better than 4 Å resolution, sufficient to fully trace the polypeptide chain and unambiguously resolve important functional regions of these molecular machines.
- Recent high-resolution structural studies of kinesins with different functionalities in complex with microtubules and tubulin protofilaments have illuminated how microtubule binding activates the kinesin ATP hydrolysis cycle, how the two motor domains of plus-end directed motile kinesins coordinate their mechanochemical cycles, how structural adaptation within the motor domain confers motile and/or microtubule depolymerization activities to different kinesins, and how accessory proteins and elements outside the motor domain regulate kinesin activity. The closure of the kinesin nucleotide-binding pocket does not depend solely on ATP binding. This step is also controlled by the position of the neck-linker in motile kinesins and by the loop-2 latch in microtubule depolymerase kinesins.
- It is expected that high-resolution studies of kinesin complexes, particularly by cryo-electron microscopy, will continue to play a key role in revealing the mechanisms of action and regulation of different kinesins. Currently, only a subset of kinesins in complex with their microtubule tracks and binding partners has been investigated at high-resolution, but the kinesin superfamily exhibits astounding variation in functionality, suggesting that more structural adaptations will be found in other kinesins. Furthermore, the achieved resolution thus far, as well as anticipated improvements in the resolution of kinesin–microtubule complex structures, will enable the use of these complex structures in structure-guided approaches to discover novel therapeutics for the treatment of kinesin-related pathologies.

## Open Access

Open access for this article was enabled by the participation of *Albert Einstein College of Medicine* in an all-inclusive *Read & Publish* agreement with Portland Press and the Biochemical Society.

## Abbreviations

ANP: AMPPNP: Adenylyl-imidodiphosphate
AAF: ADP-AlFx: Adenosine 5′-diphosphate and aluminum fluoride
CC1: coiled-coil regions
KBP: kinesin binding protein
NC: neck coil
NPO: nucleotide pocket openness
